# The Estrogen Receptor Joins Other Cancer Biomarkers as a Predictor of Outcome

**DOI:** 10.1155/2013/479541

**Published:** 2013-10-07

**Authors:** Kimberly K. Leslie, Kristina W. Thiel, Henry D. Reyes, Shujie Yang, Yuping Zhang, Matthew J. Carlson, Nirmala S. Kumar, Donghai D. Dai

**Affiliations:** Department of Obstetrics and Gynecology, University of Iowa Hospitals and Clinics, 200 Hawkins Drive, Iowa City, IA 52242, USA

## Abstract

Endometrial cancer, the most common gynecologic malignancy in the United States, is on the rise, and survival is worse today than 40 years ago. In order to improve the outcomes, better biomarkers that direct the choice of therapy are urgently needed. In this review, we explore the estrogen receptor as the most studied biomarker and the best predictor for response for endometrial cancer reported to date.

## 1. Endometrial Cancer as a Hormonally Regulated Disease

Endometrial cancer is the most common gynecologic malignancy in the United States, with an estimated 47,130 cases and over 8,500 deaths expected in 2013. The disease is on the rise and, unlike cancers arising in most other sites, five-year survival is worse today than in 1975 (87% in 1975–77; 83% in 2003–08) [1]. Biomarkers that can be used to guide treatment selection are urgently needed in order to address this alarming trend of decreasing survival. The purpose of this paper is to review the most consistently studied marker for response to therapy on clinical trials in endometrial cancer, the estrogen receptor (ER), and to highlight new information linking its expression to the outcomes.

**Figure 1:**
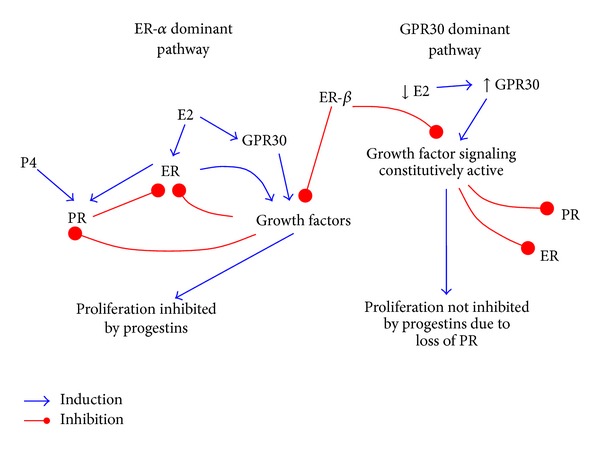
Hormone receptors in endometrial tumors. In ER-*α* dependent tumors (left side), estrogen induces growth factors and PR through ER-*α*. This creates a positive feedback loop between ER-*α* and growth factor signaling. However, progesterone (P4), when bound to PR, downregulates ER and PR. In addition, MAPK activation downstream of growth factor signaling results in phosphorylation of ER and PR and the ligand-dependent loss of PR and ER proteins by ubiquitination-mediated proteasomal degradation. ER-*α* and PR levels are increased again at the level of transcription by estrogen stimulation. Hence, the growth of these tumors is dependent upon estrogen and is limited by progesterone, suggesting that the patient will respond to progestin hormonal therapy. High expression of ER-*β*, if present, can inhibit the function of ER-*α*. For GPR30 dependent tumors (right panel), we hypothesize that proliferation is driven by the constitutive activation of one or more components of a growth factor pathway. Growth does not depend upon the presence of estrogen and is not limited by progesterone. Also, the classical steroid hormone receptors are downregulated as a result of constitutive phosphorylation via MAPK. This is predicted because the phosphorylation of the receptors leads to its targeting the proteasome for degradation. By virtue of the constitutive activation of a growth factor pathway, such tumors grow independently of classical hormonal signaling.

Estrogen binds to at least three major classes of receptors, ER-*α*, ER-*β*, and GPR30 (Figure 1). ER-*α* predominates in the endometrium and is the best studied of the three. 17 *β*-OH-estradiol is the most active ligand and, upon binding to ER-*α*, causes the transactivation of numerous growth-promoting genes, including growth factors such as epidermal growth factor (EGF) and its receptor (EGFR), insulin-like growth factor-1 (IGF-1), and growth-enhancing protooncogenes such as c-fos and c-myc [2–13]. Most relevant for this discussion are growth factors such as EGF, vascular endothelial growth factor (VEGF), fibroblast growth factor (FGF), and IGF. These ligands, in turn, activate cognate growth factor receptors, leading to multiple signaling cascades which drive cellular proliferation.

ER-*α* is induced in estrogen-driven tumors, typically grade 1 and 2 lesions, and is associated with surrounding endometrial hyperplasia. The etiology of such tumors is clearly linked to overexposure to estrogen in the absence of progesterone, the principal differentiating hormone which downregulates ER expression and counters its actions on multiple levels. Such tumors are on the rise in obese postmenopausal women, where adipose tissue produces estrone which is readily converted to estradiol in the endometrium, and are also a concern in younger women who do not ovulate due to PCOS. Such tumors have been classically referred to as type I lesions [14].

Type I endometrial cancer is of endometrioid morphology, is preceded by endometrial hyperplasia, and comprises approximately 80% of sporadic tumors. On a molecular level, type I cancers have been linked to mutations or downregulation of *PTEN*, among other targets, leading to constitutive activation of Akt and mTOR [15–18]. New data from the Cancer Genome Atlas (TCGA) also categorize endometrial tumors into multiplatform subtypes based on mRNA expression, somatic copy number alterations, microsatellite instability (MSI), and somatic nucleotide substitutions [19]. These data confirm that high ER-*α* expression (ESR1) is a characteristic of lower grade tumors which are also associated with mutations in *PTEN* (the “copy-number low” cluster). Moreover, RNA-seq and reverse phase protein array (RPPA) data demonstrate that ER-*α* mRNA and protein expression and phosphorylation on Ser118, indicating its activity, strongly correlate with the PTEN-null, MSI hyper-mutated, and copy-number low cluster which is also associated with E-cadherin expression and activation of polycystic kidney disease 1 (PDK1) and Akt [20]. Exome sequencing has also revealed a high prevalence of mutations in *ARID1A *[20, 21], which likely contributes to PI3K activation in the “copy-number low” cluster.

In comparison, type II tumors comprise a heterogeneous, poorly differentiated group of tumors of high grade endometrioid, serous papillary, or clear cell morphology that primarily occurs in older postmenopausal women. Type II cancers are well known to harbor mutations in *TP53* and demonstrate higher expression of *ErbB2* [18, 22–24]. These tumors are often locally advanced and/or metastatic, and they carry a very poor prognosis [25]. For such lesions, survival is often less than six months despite aggressive chemotherapy and radiation. The TCGA confirms the general categorization of type II lesions to include serous, serous-like, and a subset of endometrioid tumors, mostly of high grade, which make up approximately 25% of all type II tumors when segregated based upon genomic data. Again, the strong correlation of *TP53* mutations, resulting in aberrant protein expression, is noted in this “high copy-number cluster” TCGA subtype. CHK2 phosphorylation on T68 and the high expression of cell cycle regulators, Cyclin E, Cyclin D, and CDK1, are characteristics of these tumors [19]. In addition, genes involved in chromatin remodeling and ubiquitin ligase complexes are frequently mutated in serous tumors [26, 27]. RPPA and RNA-seq data demonstrate that PTEN expression is present, and ER-*α* expression is generally low [20]. However, it is possible that other estrogen receptors are present in type II tumors, and further analysis of the TCGA and other datasets should shed light on this question, as discussed below.

ER-*β* expression, though lower than ER-*α* in most endometrial cancers, may be induced in some tumors, in particular endometrial tumors of a higher grade [28]. Reports suggest that it may inhibit the function of ER-*α* and/or that it may be a marker for poor outcome [29, 30]. However, these data are complicated by the presence of several ER-*β* splice variants that are differentially associated with tumor grade [28].

A novel intracellular seven-transmembrane G protein-coupled estrogen receptor (GPR30) appears to function alongside the traditional estrogen receptor to regulate physiological responsiveness to estrogen and is now considered a new estrogen receptor [31]. GPR30 has also been linked to poor clinical outcomes in endometrial cancer patients [32]. GPR30 signals through EGFR to control PI3K and MAPK activity [33, 34]. In turn, these phosphorylate ER and progesterone receptor (PR), resulting in their degradation in the proteasome. In addition to estrogen, classic ER antagonists such as tamoxifen activate multiple cellular signaling pathways via GPR30 [31, 34, 35]. Partial or biologically weak estrogens can also activate ER-*β* to a greater degree than ER-*α*, indicating that these receptors may be functional despite low levels of estradiol. In conclusion, the three different receptors for estrogen appear to segregate between a classic versus an alternative, or GPR30-driven, pathway, as shown in Figure 1.

## 2. ER-*α* and PR Markers for Sensitivity to Hormonal Therapy

The expression of ER and PR is linked because transcription of the PR gene is induced by estrogen and inhibited by progestins [36, 37]. Data from the Gynecologic Oncology Group (GOG) Core Laboratory for Receptors have shown that ER-*α* on a pretreatment biopsy predicts response to hormonal therapy in GOG study 119, tamoxifen and intermittent medroxyprogesterone acetate for advanced endometrial cancer [38]. These data were more recently confirmed in a preliminary analysis of GOG study 248, where hormonal therapy with tamoxifen and intermittent progestin with an mTOR inhibitor was compared to the mTOR inhibitor alone. A general theme from these studies is that ER-*α* expression correlates with PR expression, but interestingly, the correlation differs somewhat by PR isoform. ER was most strongly associated with PRA expression compared to PRB. The implications of this finding with respect to endometrial carcinogenesis and progression are substantial, given the different functions of the PR isoforms, and should be validated in future clinical trials. Indeed, the importance of identifying PRA compared to PRB has been assessed. Our laboratory published on the expression of the isoforms in well-differentiated compared to poorly differentiated endometrial cancer cell lines and showed that loss of PRB is associated with loss of differentiation [25].

The requirement of PR for endometrial function, secretion, and immunomodulation, as well as limiting the proliferative effects of estrogen, has been well documented [39–43]. The use of PR and ER as markers of response to therapy is generally supported by the literature [38], yet unfortunately in our view, the receptors are not routinely assessed in endometrial cancer specimens. Perhaps the introduction of more effective hormonal regimens, whereby PR expression is enhanced and maintained by epigenetic modulation, will provide new opportunities to treat patients with endometrial cancer [44, 45]. With improved hormonal regimens on the horizon, the assessment of tumors for receptor expression will become even more imperative.

## 3. Molecular Inhibitors and ER

In addition to hormonal therapy, targeted treatments are also used for advanced endometrial cancer [46–50]. Somewhat surprisingly, ER-*α* has been the most consistent and robust marker for overall survival (OS) in patients on these trials. The GOG has studied a number of agents in the 229 queue, including gefitinib (229C), lapatinib (229D), bevacizumab (229E), and brivanib (229I). One explanation for the finding that ER-*α* positively correlates with OS is that such tumors are better differentiated and less aggressive. However, even after controlling for stage and grade, ER-*α* remains a predictive marker. An alternative explanation is that each of these inhibitors blocks an estrogen-induced growth factor pathway (EGF, Her-2, VEGF, and FGFR). Tumors with high ER-*α* expression, which have developed in the setting of estrogen excess, are reliant on estrogen-driven pathways for survival and are the most responsive to treatment when such pathways are blocked. This hypothesis should be further evaluated in future studies of molecular inhibitors which impact growth factors downstream of ER-*α*.

## 4. Conclusion

Despite the predictability of ER-*α*, its expression has not been clinically evaluated in the routine care of patients with endometrial cancer. We propose that ER-*α* should be recognized as a biomarker for positive outcome in endometrial cancer and its presence assessed on patient specimens. Immunohistochemistry (IHC) is the most appropriate methodology to measure ER-*α* from clinical samples due to feasibility (general lack of access to fresh frozen tissue) and the long track-record of IHC as a reliable measure of ER-*α* expression. Singh et al. (GOG119) serves as the basis for determining ER-*α* expression in primary tumor tissue by IHC [38].

ER-*α* is predictive of positive outcomes in endometrial cancer, both OS in general and to therapy. It is a marker for a hormone responsive tumor, and such cases should be considered for hormonal therapy. In addition, tumors with high ER-*α* expression are also dependent upon downstream growth factor signaling and may respond better to molecular inhibitors of the EGF, VEGF, and FGF pathways.

While other cancer biomarkers such as PSA and CEA are negative markers which indicate the presence of cancer or its recurrence, ER-*α* is a positive marker for better clinical outcomes in women with endometrial malignancy. The usefulness of a positive biomarker may not be intuitively obvious. However, we propose that positive biomarkers can be helpful in directing therapy to agents with a higher potential of improving outcomes, that is, hormonal treatment and specific targeted agents, allowing ER-*α* negative tumors to be treated by other means such as adjuvant chemotherapy. While the impact of the other forms of estrogen receptors (GPR30 and ER-*β*) on outcomes deserves further study, it is clear that ER-*α* is a confirmed biomarker. ER-*α* positive tumors are more likely to be cured with hysterectomy alone. Such cases may require no additional treatment, a hypothesis which should be tested prospectively in future studies. Limiting excessive therapy may provide substantial benefit to patients and is an important goal which may be positively impacted by the use of ER-*α* as a biomarker.
